# Quantitatively Verifying the Results' Rationality for Farmland Quality Evaluation with Crop Yield, a Case Study in the Northwest Henan Province, China

**DOI:** 10.1371/journal.pone.0160204

**Published:** 2016-08-04

**Authors:** Yali Zhang, Junchang Huang, Lin Yu, Song Wang

**Affiliations:** 1 College of Resources and Environment, Henan Agricultural University, Zhengzhou, China; 2 Engineering Research Center of Agricultural Resources and Environment, Colleges and Universities of Henan Province, Zhengzhou, China; University of Delhi, INDIA

## Abstract

Evaluating the assessing results’ rationality for farmland quality (FQ) is usually qualitative and based on farmers and experts’ perceptions of soil quality and crop yield. Its quantitative checking still remains difficult and is likely ignored. In this paper, FQ in Xiuwu County, the Northwest Henan Province, China was evaluated by the gray relational analysis (GRA) method and the traditional analytic hierarchy process (AHP) method. The consistency rate of two results was analysed. Research focused on proposing one method of testing the evaluation results’ rationality for FQ based on the crop yield. Firstly generating a grade map of crop yield and overlying it with the FQ evaluation maps. Then analysing their consistency rate for each grade in the same spatial position. Finally examining the consistency effects and allowing for a decision on adopting the results. The results showed that the area rate consistency and matching evaluation unit numbers between the two methods were 84.68% and 87.29%, respectively, and the space distribution was approximately equal. The area consistency rates between crop yield level and FQ evaluation levels by GRA and AHP were 78.15% and 74.29%, respectively. Therefore, the verifying effects of GRA and AHP were near, good and acceptable, and the FQ results from both could reflect the crop yield levels. The evaluation results by GCA, as a whole, were slightly more rational than that by AHP.

## Introduction

The soil quality discussion that has developed since the 1970s has raised important issues about soil assessment and management practices in many countries [1,2,3]. The Food and Agriculture Organization (FAO) of the United Nations [4], the United States Department of Agriculture [5], and the European Union [6] all identified soil quality as a work focus and used soil quality evaluation as an important index for assessing soil quality changes and to promote land management. At the same time, soil quality evaluation is often frustrating due to the lack of direct testing of the proposed methods and assessment results. Because the different evaluation indicators are determined for different evaluation purposes and different soil functions, there is no unified international evaluation standard and method of soil quality [7,8]. The usual evaluation methods can be summed up as ranking methods [2, 5] and parameter ones, based on qualitative and quantitative assessment [9,10], respectively. Compared with ranking methods, the parameter methods reflect the precise relationship between land productivity and the parameter measured, and can reduce the subjective effect from artificial factors and interferences. Therefore, these methods always obtain more accurate evaluation results and are widely used, including the soil quality index method [11,12], soil quality function model [13,14], soil quality dynamics model [15,16], multiple linear regression model [3,17], and relative soil quality model [18], etc.

In China, soil quality evaluation mainly focuses on farmland quality (FQ) and is generally divided into two categories. One is the currently productive capacity evaluation based on the crop production, and the other is the farmland potential one mainly based on natural elements, such as soil physical and chemical properties, landform, rainfall and evaporation, irrigation and drainage, etc. [19]. The Ministry of Agriculture of the People's Republic of China (MOAPRC) has nationally carried out FQ assessments of potential productive capacity since 2002 [20]. FQ evaluation in China is mostly performed using geographic information system (GIS) technology [19,20]. The main methods include the analytic hierarchy process (AHP) [21], the fuzzy comprehensive evaluation (FCV) method [22], a principal component analysis (PCA) [23], regression analysis (RA) methods [24], the Delphi method [25], and the gray correlation analysis (GCA) method [26], etc.

According to the Rules for Farmland Quality Survey and Assessment [27], the main process is based on AHP and Delphi methods, and the determination of indices’ weight depends on the experience of individual experts. Thus, there is inherent subjectivity in ascertaining the importance of indices. Meanwhile, the cross influence among these indexes is also difficult to judge [21]. Because FQ evaluation is a complex system, the evaluation indices are not independent from each other and the relationship between them is not definite [19], it should be a grey correlation variant [26]. In determining the weight of FQ indices, GCA is more objective than AHP [28]. Therefore, to reduce the interference from human factors, some researchers suggested that GCA should be embedded in FQ evaluation [26,29]. Furthermore, to improve the objectivity of the FQ evaluation, it is urgent to compare and analyse the different evaluation results by AHP and GCA as soon as possible.

Due to the divergence of experts’ opinions and the limitations of mathematical methods, there is an inevitable deviation in the evaluation results, especially in the first round [19,20]. Therefore, verifying the evaluation results can greatly improve FQ evaluation and promote its practical application. FQ levels can represent practical productivity to a great extent. In other words, there is a correlation between crop yield and FQ levels. Therefore, crop yield can be used as reference to validate the results [8,23,30]. In evaluating soil fertiliser, farm yard manure, and crop management practices on a semiarid inceptisol in India, Masto et al. (2008) [31] developed a sensitive soil quality index and compared the sensitivity index with crop yields. Li et al. (2013) [24] reported a significant positive correlation between soil quality index (SQI) and rice yield. Liu et al. (2015) [8] further described a linear relationship between SQI and crop yield using correlation analysis and found a correlation coefficient of 0.755. Mueller et al. (2013) [32] proposed that more than 70% of crop yield variability at a given input intensity can be explained by the soil quality, which includes complex information. In China, FQ assessment results’ test often depends on the experience or sense of local farmers and agricultural management [19,20], a factor which is ignored at present. Compared with the information available on FQ evaluation methods and processes [14,25] and the relationships between soil indicators and crop yield [8,31,33–35], the reports on the test of FQ evaluation level are incomplete [23]. Therefore, the main objectives of this study were to compare the different FQ evaluation results from AHP and GCA and to develop one quantitative method to test their rationality.

## Materials and Methods

### Study site

**Fig 1:**
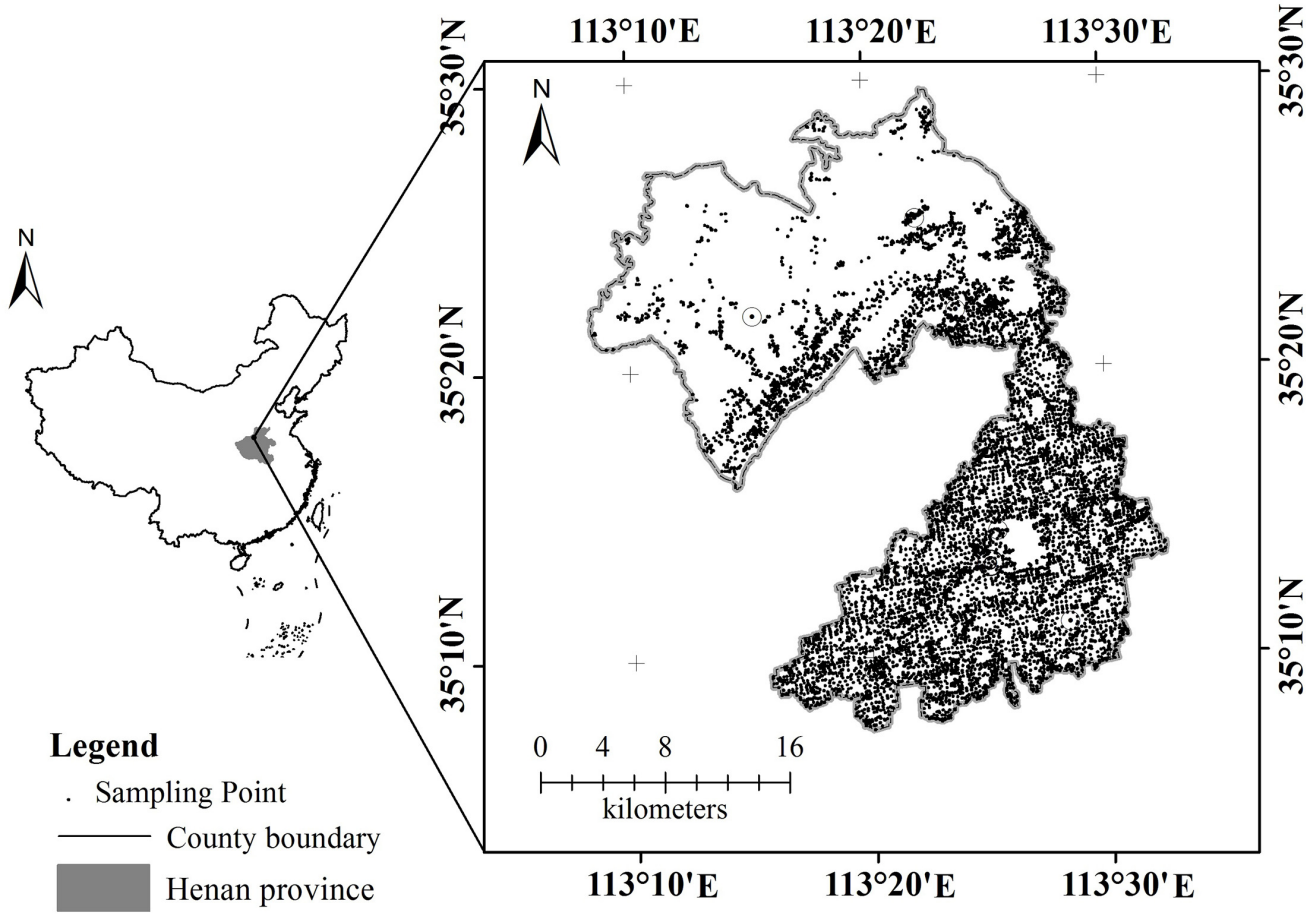
Geographic location of study region and soil sampling sites.

The study was carried out in Xiuwu County, Henan Province, China, which is located in the northwest Henan Province and south of Taihang Mountains, as shown in Fig 1. According to the Xiuwu County Annals [36], the north is mountainous area and hills, and the south is alluvial plains with an altitude ranging from 77.4 to 1308 m above sea level. The mean annual rainfall is 560.4 mm, and the mean annual temperature is 14.5°C. The average annual frost-free period is 216 days, and the average annual sunshine period is 2062.4 hours. The county has 27,000 ha of farmland and a per capita farmland area of 0.089 ha. The major soil types are brown soil, brown loam, tidal soil and saline soil, with the former two being the most soil types with higher crop output.

### Basic data and analytic approach

There were four types of data included in the evaluation. The first type was the information on the sampling points surveyed, such as geographic and geomorphic conditions, soil parent material, the thickness of planting soil layer, soil texture, farmland utilisation, irrigation and drainage, crop yield and land management. The second type was the soil analysis data from the sampling points, including all the soil nutrient content, pH and organic matter content, etc. The third type was the local social and economic statistical data, land area, standard farming system, the local crops (both grain and cash crops), and agricultural investment. The last type was basic maps at a 1:50 000 scale, including a soil type map, land-use map, soil nutrient map, topographic map, and the latest administrative map, etc. [19,20]. There were a total of 1993 soil samples and the sampling points in the study region were shown in the Fig 1. The data analysis approach was briefly described here [27]. The former three dataset’s attributes were chosen, classified and reorganised in Excel software. Then, the attribute databases of soil nutrient and soil type, etc. were set up in Access software. For the map data, the maps were scanned, digitised, graphically edited, error corrected, and topology amended using MapGIS 6.7, and then the data were stored in spatial databases. Last, the spatial databases were associated with the attribute databases using the position key field, and the basic database information of FQ was founded.

### Evaluation methods

The FQ evaluation units were generated by overlaying the digital soil map with the land-use map. Each small patch less than the map area of 4 mm^2^ and less than actual area of 0.04 km^2^ was merged to create a new unit. The FQ evaluation units in the study area were divided into a total of 7779 units. The units were analysed, and the evaluation indexes were chosen based on recommendations from local experts. In total, 10 total indices were chosen, including soil organic matter, available phosphorus in the soil, available potassium in the soil, landform, soil texture, soil texture configuration, soil section configuration, soil barrier layer position, arable layer salinity and irrigation assurance, etc. The value of each index in the evaluation units was extracted from the previously described databases. The quantification of each evaluation index was calculated according to the published methods [19,20,27]. Quantitative indicators for the soil organic matter and available phosphorus and potassium in the soil were respectively calculated using the function models between each of them along with the land fertility [19,20]. For qualitative indicators, such as landform, soil texture, soil texture configuration, soil section configuration, soil barrier layer position, arable layer salinity and irrigation assurance rate, the quantification was an average value by the Delphi method [27].

The calculation process of evaluation index weight by GCA was as follows. First, a survey of the standard farming system and the local crops, both grain and cash crops, was included. By assigning each administrative village of research area one survey point, we surveyed the annual crop yield over the last 3–5 years. Cash crop yield was counted based on wheat or rice as the standard crop and the productive ratio coefficient *β*_*i*_ as shown in formula 1 [37], so that the equivalent yield of all of the crops could be found by multiplying the surveyed yield by *β*_*i*_. Thus, the total annual yield for the standard farming system was calculated.

$$\beta_{i}=\frac{Q_{\text{max}}}{q_{\text{max}}}$$ (1)

Where *β*_*i*_ was the productive ratio coefficient of one special crop, Q_*max*_ was the maximum yield of the standard crops, kg.ha^-1^, and q_*max*_ was the maximum yield of the other special crop, kg.ha^-1^.

Then, the total annual yield in each evaluation unit was determined as the reference sequence {x_0_}. After quantifying the evaluation indices, the membership grade was assigned to each evaluation unit as a comparative sequence {x_i_} (i = 1, 2, 3… n). According to the relational model of grey system theory, we calculated the correlation coefficient (*L*_0*i*_*(k)*) between the two sequences over time, t = k, using formula 2 [28].

$$L_{0i}(k)=\frac{\underset{i}{\text{min}}\;\underset{k}{\text{min}}\;\left|x_{0}(k)-x_{i}(k)\right|+\;\rho \;\underset{i}{\text{max}}\;\underset{k}{\text{max}}\left|x_{0}(k)-x_{i}(k)\right|\;}{\left|x_{0}(k)-x_{i}(k)\right|+\rho \;\underset{i}{\text{max}}\;\underset{k}{\text{max}}\left|x_{0}(k)-x_{i}(k)\right|\;}$$ (2)

Where *ρ* was the resolving coefficient (0<*ρ*<1), and *ρ* = 0.5.

The correlation degree (*R*_0*i*_) was calculated using formula 3 [28].

$$R_{0i}=\frac{1}{n}\sum_{k=1}^{n}L_{0i}(k)$$ (3)

Then, the evaluation index weight (w_i_) was calculated using formula 4 [28].

$$W_{i}=\frac{R_{0i}}{\sum_{i=1}^{n}R_{0i}}(i=1,2,3,\cdot \cdot \cdot n)$$ (4)

For the AHP method, the weights were determined by the Delphi method based on the MOAPRC and previous research [20,24,25]. The soil quality index (SQI) for each evaluation unit was calculated using formula 5 for each of the two methods.

$$SQI=\sum_{i=1}^{n}S_{i}\times W_{i}$$ (5)

Where S_i_ was the quantisation score of each evaluation index, and n is the number of variables. Last, the FQ grades for the evaluation units were defined by K-homogeneity Cluster Analysis of their SQI [20,27]. The number of FQ grades, no more than 10 in total, was based on the divergence of average crop yield [19,27].

### Determination of crop yield grade

According to statistical principles, when the survey samples reach a certain number, the statistical yield would gradually converge on the average in a specified area (such as a village, a township or a county) or with a particular soil type. We defined it as the conceptual yield [20]. If actual yield data of the soil sampling in some years was present, the conceptual yield was recommended as a test sample. If not, it was recommended to study the local agricultural investment, management level and climate for a long series years, and to choose one as the test sample, whose actual crop output was approximate to that of the recent years, and would be steady to a certain extent. According to the *Classification of Type Regions and Quality of Farmland in China* [38], because of their different productive capacity and crop yield per unit area, the national FQ was divided into 10 levels. The food yield per unit area from 1^st^ to 10^th^ was an arithmetic progression with 1500 kg ha^-1^ common difference, where the 1^st^ level was more than 13500 kg ha^-1^ and that of 10^th^ was less than 1500 kg.ha^-1^. During actual evaluation, the number of yield grades could be less than 10 due to local crop yield, which should be similar to the FQ levels. Each evaluation unit was assigned a grade based on the total annual yield. The yield grades of the whole region could be predicted by fractal kriging interpolation with ArcGIS 9.3.

### Rationality check of the FQ evaluation results

The yield grade map and the FQ grade maps created using the previously described two methods were overlaid and topologically analysed for the area consistency and spatial distribution of each grade. Due to the lack of previous research on a rational standard to judge FQ evaluation results, the area consistency rate (ACR) was used as a criterion in evaluation. ACR was the percentage of each grade area in the FQ map divided by that of the same grade in the yield map in the same spatial site. The FQ here evaluates the potential production of farmlands, while the crop yield grade is based on actual production [26,30]. Therefore, combining the practical FQ evaluation, the advice of agricultural experts and the research of Mueller et al. (2013) [32] and Liu et al. (2015) [8], if the total ACR>70, the evaluation result was considered to be good or excellent and was both reasonable and acceptable (Table 1). Otherwise, to reach this acceptable level of evaluation, we should re-evaluate the FQ and re-check the ACR.

**Table 1:** Rationality test of the area consistency rate between the FQ evaluation grades and the crop yield grades.

ACR ^a^ %	ACR≤50	50<ACR≤60	60< ACR≤70	70< ACR≤85	85<ACR≤100
Test effect ^b^	Bad	Poor	Medium	Good	Excellent
Acceptable level ^c^	Unacceptable			Acceptable	

^a^ ACR was Area consistency rate, which could be calculated from the percentage of the area in the FQ map divided by the equivalent level in the yield grades map.

^b^ Test effect indicated the consistency degree between the FQ evaluation grades and the crop yield grades.

^c^ Acceptable level was according to the test effect and allowed for a decision on adopting the results or abandoning them.

Abbreviation: FQ, Farmland Quality.

## Results and Discussion

### FQ evaluation results based on GCA

**Fig 2:**
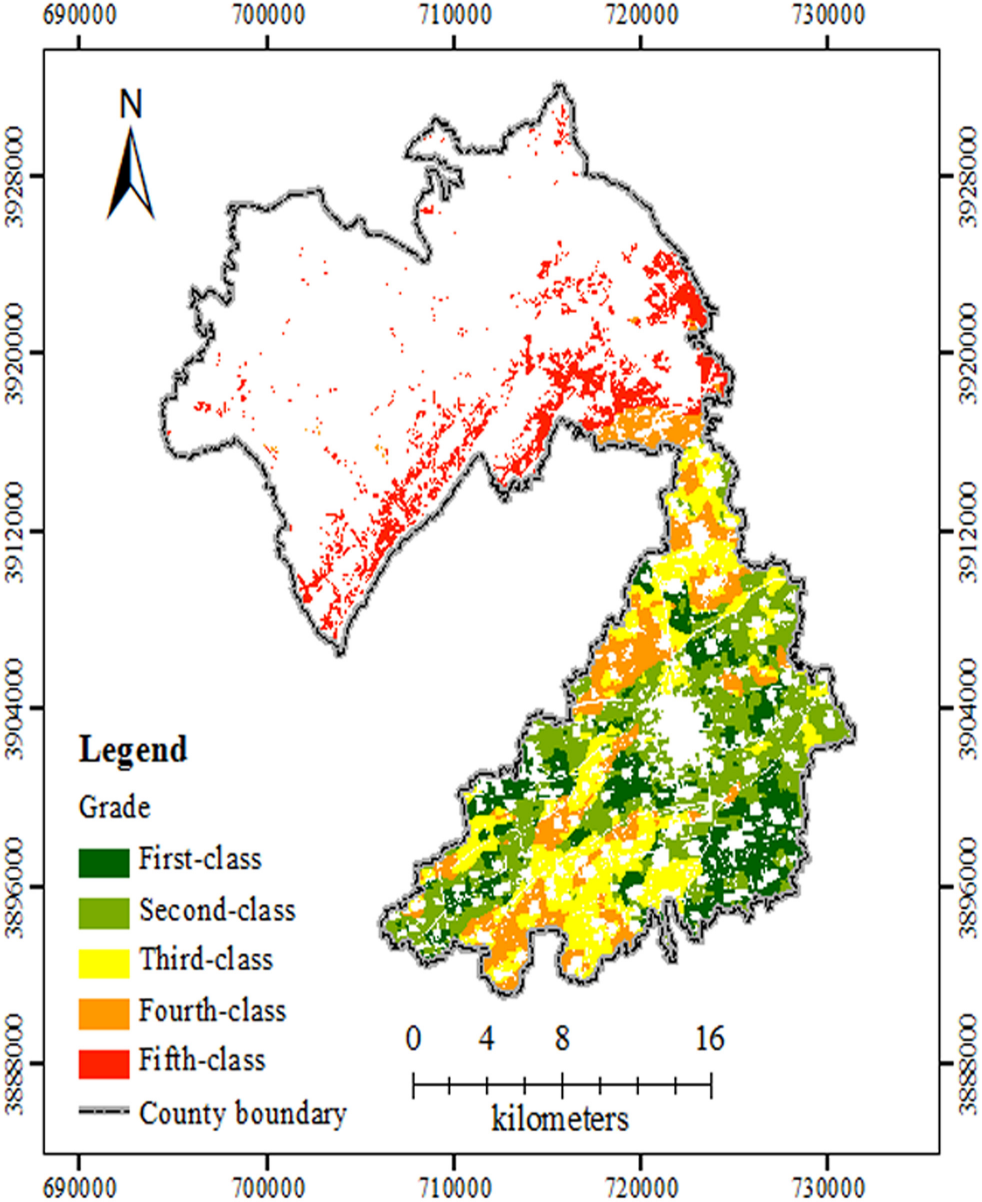
Level distribution map of FQ evaluations based on GCA in the study site. The different grades of FQ evaluations were shown with different colours. Abbreviations: FQ, Farmland Quality; GCA, gray correlation analysis.

The standard farming system in Xiuwu County is double cropping in one year, and the main crops are wheat and maize [36]. The annual crop yields from 2005 to 2010 for each administrative village within the study area were investigated. Wheat was considered the standard crop, and its yield was determined. Using formula 1 [28], the maize yield was converted to match wheat, and then the total annual yield was calculated. Last, the ten evaluation index weights were calculated using the GCA method (Table 2). The SQI of all of the evaluation units using the GCA method was between 0.5617 and 0.9651. According to the previous research [20,22] and the advice from local experts, five FQ grades was an acceptable number for the complex landform in the study region. The first-class farmland is the best followed by progressive decreases to second-class, third-class, fourth-class and fifth-class. According to the cluster analysis results, the area from the first to fifth classes had 4670 ha, 7574 ha, 5952 ha, 4611 ha and 3951 ha, respectively. As shown in Fig 2, the first-class and second-class farmland was mainly located in the southern plains, with fertile soil, available irrigation water and convenient traffic. The inferior third-class, fourth-class and fifth-class farmland were in the northern mountainous and hilly areas, which had high altitudes, poor transportation, few irrigation facilities, and broken or infertile farmland.

**Table 2:** Correlation degree and weight of FQ evaluation indexes based on GCA in study site.

Evaluation index	Correlation degree ^a^	Weight value ^b^	Evaluation index	Correlation degree ^a^	Weight value ^b^
Soil organic matter	0.5688	0.1056	Irrigation ensuring rate	0.7353	0.1365
Available potassium in the soil	0.3970	0.0737	Soil section configuration	0.5015	0.0931
Available phosphorus in the soil	0.4816	0.0894	Soil barrier layer location	0.5203	0.0966
Landform	0.6001	0.1114	Soil texture	0.5279	0.0980
Arable layer salinity	0.5462	0.1014	Soil texture configuration	0.5080	0.0943

^a^ Correlation degree represented the relationship between the annual crop yield and the evaluation index value, which could be determined using formula 3.

^b^ Weight value was calculated using formula 4.

Abbreviations: FQ, Farmland Quality; GCA, gray correlation analysis.

### Contrasting the FQ evaluation by GCA and AHP

**Fig 3:**
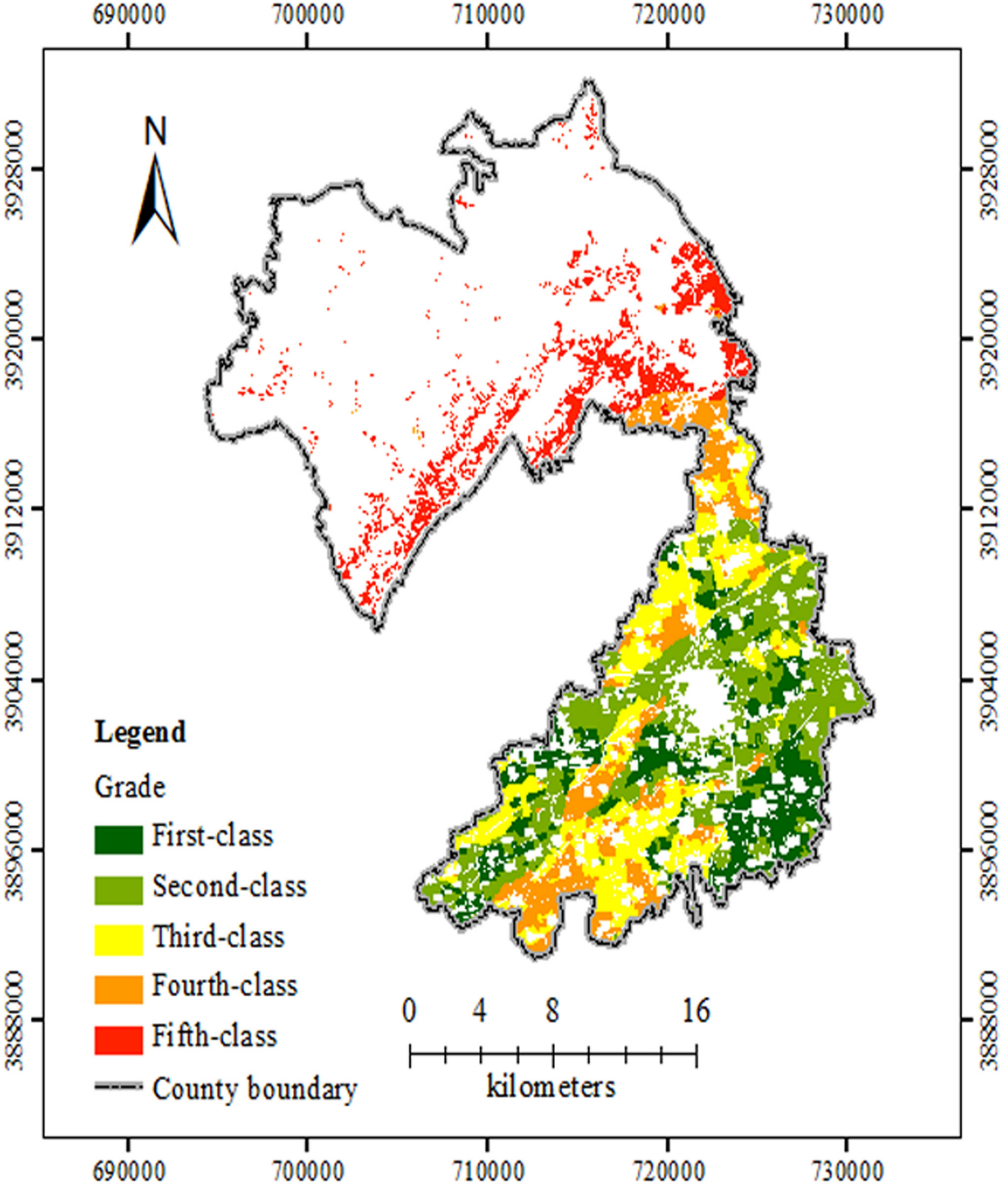
Level distribution map of FQ evaluation based on AHP in the study site. The different grades of FQ evaluations were shown with different colours. Abbreviations: FQ, Farmland Quality; AHP, analytic hierarchy process.

**Fig 4:**
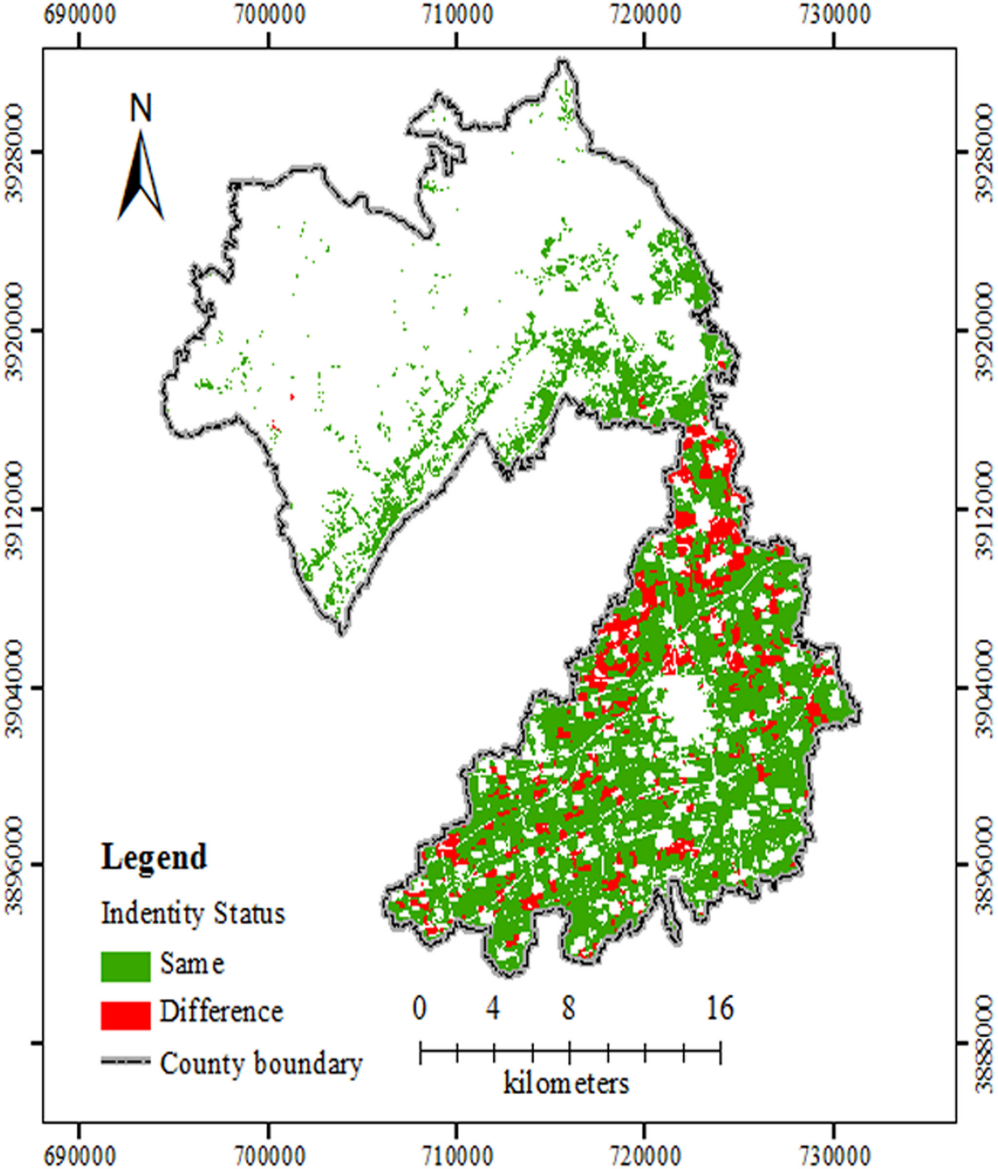
Comparison of FQ results by GCA and AHP in the study site. According to the comparison, there were two indentify status with different colours. Same indicated that FQ evaluation levels by GCA and AHP method for some unites were coincident. Difference meant that FQ levels by two methods did not well agree. Abbreviations: FQ, Farmland Quality; GCA, gray correlation analysis; AHP, analytic hierarchy process.

The FQ assessment by AHP was according to the process of published protocols [20,21,27] and was shown in Fig 3. Fig 4 was an overlay map combining Figs 2 and 3. Fig 4 and Table 3 showed that the area of each level, the proportion of each evaluation unit, and the spatial distribution, evaluated by the two methods in the research area, were nearly equivalent. For each grade in the same spatial position, the area or evaluation unit number of GCA method was divided by that with the same grade of AHP method, respectively. The percentage was consistency rate (CR), which was shown in Table 3. For the whole study region, the area or evaluation unit number with the same grade by the two methods, were divided by that of the entire farmland, respectively. The total CR for the area and evaluation unit number was 84.68% and 87.29%, respectively. It showed that the calculation procedures for index weight of the two methods varied but both were reasonable. In other way, the same evaluation indexes and quantification of the two methods also benefitted their consistency rate. These results were in accordance with those from Fengshun County, Guangdong Province [26]. The CR for the first-class, second-class and fifth-class lands was higher than those for the other 2 classes, which still needed further research.

**Table 3:** Consistency Rate (CR) analysis of matched grades based on GCA and AHP in the study site.

FQ grade	GCA		AHP		The same		CR of area (%)	CR of evaluation unit number (%)
	Area (ha)	Evaluation unit number	Area (ha)	Evaluation unit number	Area (ha)	Evaluation unit number		
The first-class	4721	1119	4670	1110	4228	1008	90.54 ^a^	90.81 ^a^
The second-class	8073	1876	7574	1755	6857	1590	90.53 ^a^	90.60 ^a^
The third-class	5712	1457	5952	1489	4271	1096	71.76 ^a^	73.61 ^a^
The fourth-class	4257	1127	4611	1247	3351	918	72.67 ^a^	73.62 ^a^
The fifth-class	3994	2200	3951	2178	3951	2178	100.00 ^a^	100.00 ^a^
Total	26758	7779	26758	7779	22658	6790	84.68 ^b^	87.29 ^b^

^a^ For each grade, CR was calculated as the percent of the area or evaluation unit number by GCA method compared to that by AHP method, respectively.

^b^ For the whole region, CR was calculated as the percent of the FQ grade area or evaluation unit number compared to the total farmland.

Abbreviations: FQ, Farmland Quality; GCA, gray correlation analysis; AHP, analytic hierarchy process.

### Mapping the crop yield grade

**Fig 5:**
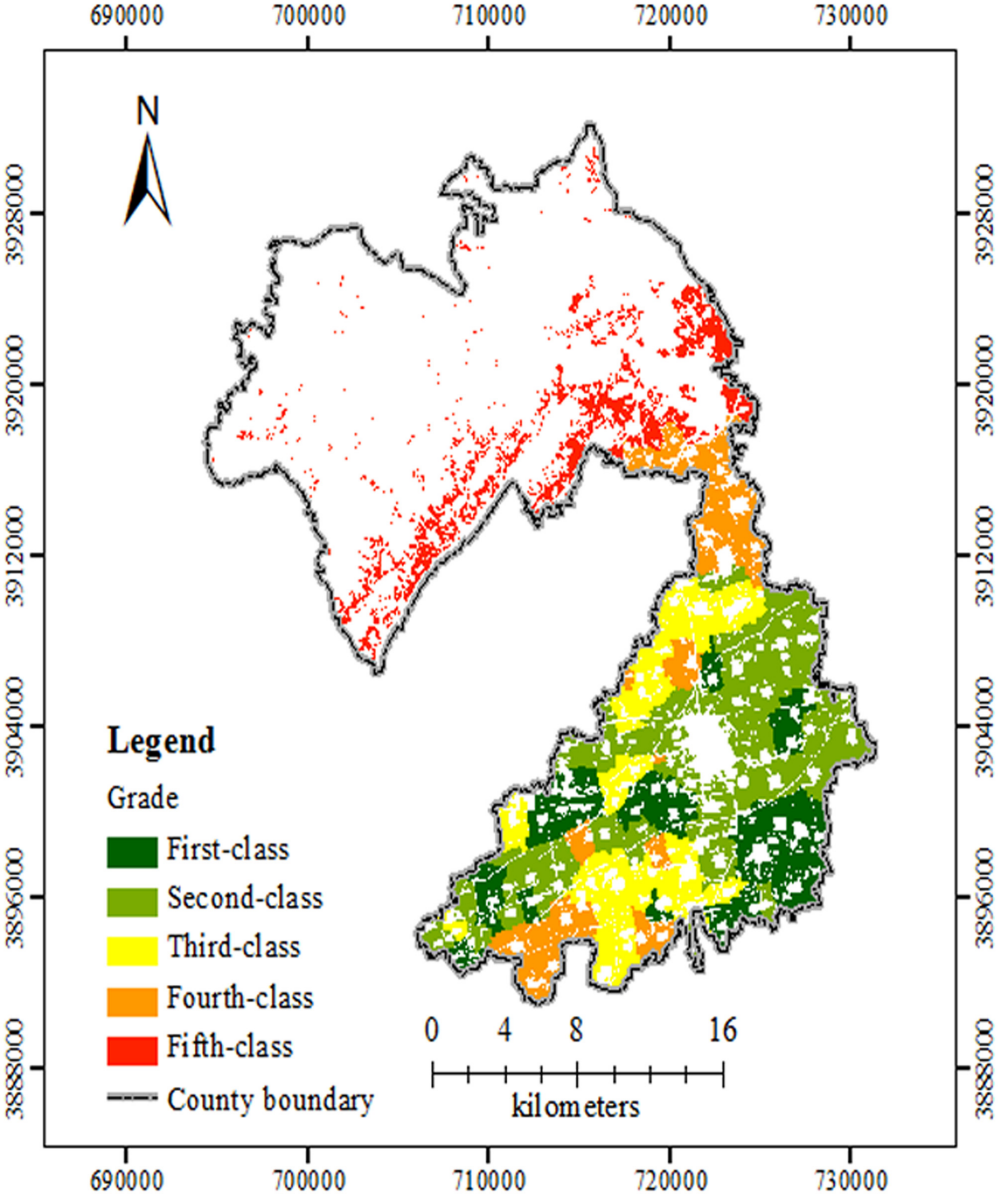
Grade distribution map of the annual crop yields in the study site. The different grades of crop yields were shown with different colours.

When determining the evaluation index weight with AHP, we did not consider the crop yield of each evaluation unit as a standard as in GCA. Therefore, to objectively test the two evaluations methods, we used the crop yield of typical year as the test sample. After statistically analysing the annual fluctuation of the county’s crop yield from 2005–2010, the agricultural capital investment, agricultural management level and meteorological conditions, the crop output (mainly of wheat and maize output) in 2010 year was selected as the test sample. According to the *Classification of Type Regions and Quality of Farmland in China* [38] and advice from the local administrators, the actual food output in 2010 year was divided into 5 levels as shown in Table 4. After kriging interpolation, the crop yield level map generated and was shown in Fig 5.

**Table 4:** Crop yields and levels in the study site.

Levels	The first-class	The second-class	The third-class	The fourth-class	The fifth-class
^a^ Crop yield (kg. ha^-1^.year^-1^)	>13,500	12,000–13,500	10,500–12,000	9,000–10,500	<9,000

^a^ The crop yield per unit area from 1^st^ to 5^th^ was an arithmetic progression with 1500 kg ha^-1^ common difference, where the 1^st^ level was more than 13500 kg ha^-1^ and that of 5^th^ was less than 9000 kg.ha^-1^.

### Rationality test of FQ evaluation results based on the crop yield grade

**Fig 6:**
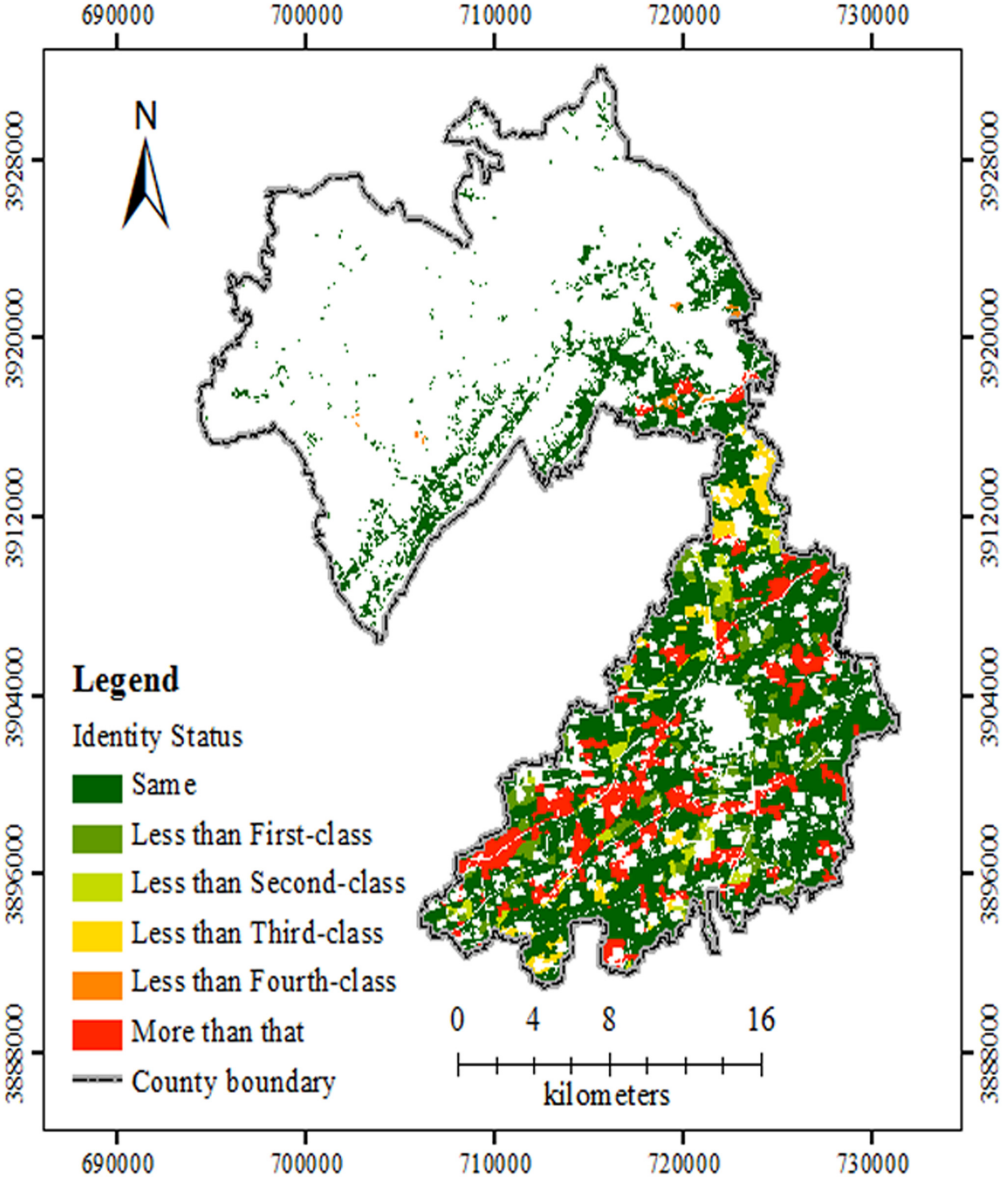
Comparison of the FQ evaluation results based on GCA and yield grades. According to the comparison, there were some indentify status with different colours. Same indicated that the FQ grade area based on the GCA methods was the same as that of the yield level. Less indicated that the grade of the former was less than that of the latter. More indicated that the former was more than the latter. Abbreviations: FQ, Farmland Quality; GCA, gray correlation analysis.

**Fig 7:**
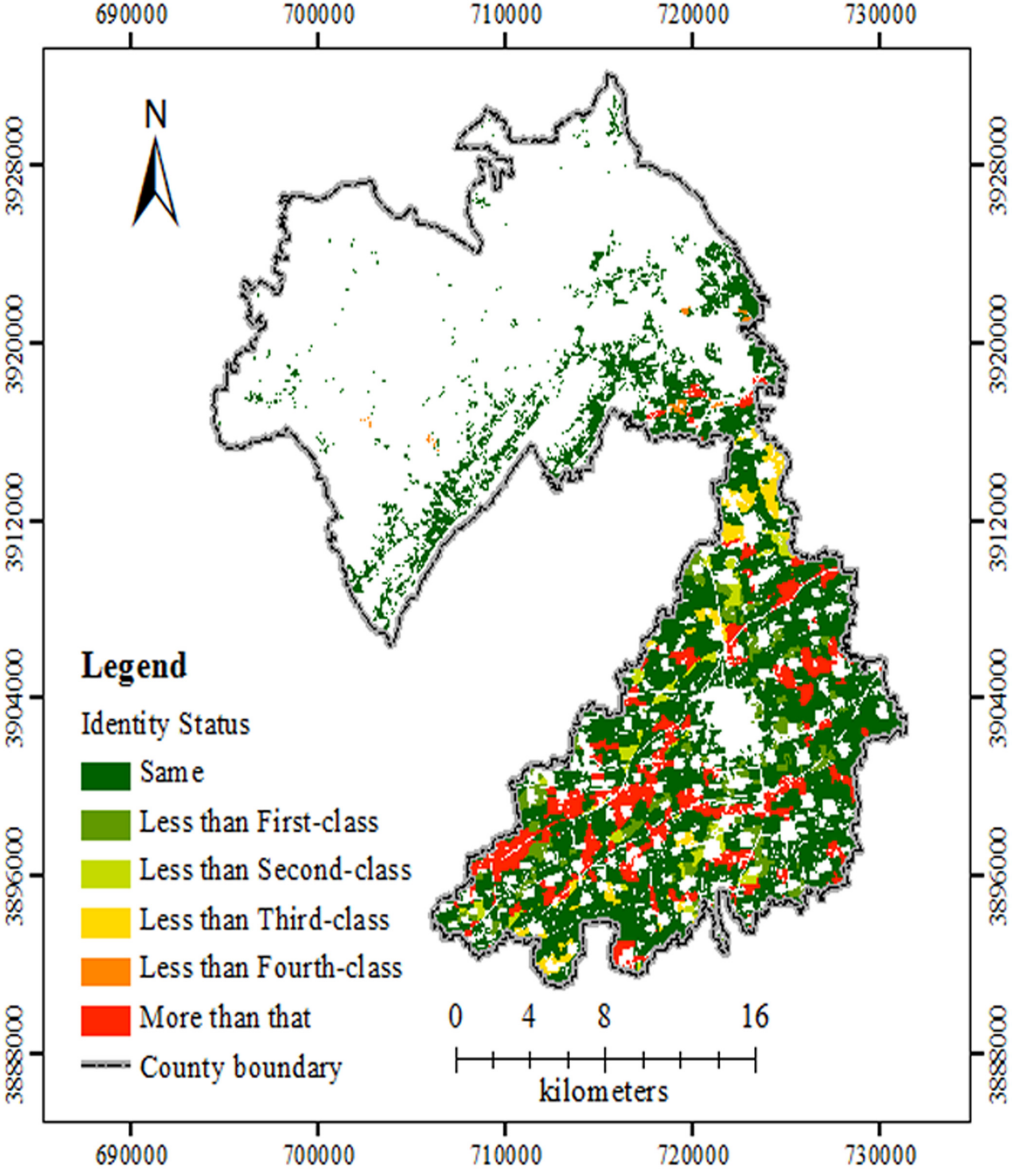
Comparison of the evaluation results based on AHP and yield grades. According to the comparison, there were some indentify status with different colours. Same indicated that the FQ grade area based on the AHP methods was the same as that of the yield level. Less indicated that the grade of the former was less than that of the latter. More indicated that the former was more than the latter. Abbreviations: FQ, Farmland Quality; AHP, analytic hierarchy process.

By overlaying the yield grade map with the FQ grade maps from both methods, we generated Figs 6 and 7. The ACR for the entire same grade by GCA method added up to 78.15%, while it by AHP method came to a total of 74.29% (Table 5). The evaluation results from GCA were slightly better than those from AHP. It is because that the GCA is good at quantitative assessments [28], while AHP is good at qualitative assessments [20]. In addition, the yield of per unit was applied in determining the weight of the FQ indices. So the GCA method is more objective and practical than APH but usually requires more yield data.

**Table 5:** Comparison of the FQ grades as evaluated by both methods with the crop yield grades.

Grade comparison	Comparison the evaluation results of GCA with crop yield grade		Comparison the evaluation results of AHP with crop yield grade	
	Area (ha)	Proportion ^a^ (%)	Area (ha)	Proportion ^a^ (%)
FQ grade was more than yield grade				
The first-class	1364	5.10	1582	5.91
The second-class	717	2.68	683	2.55
The third-class	971	3.63	1097	4.1
The fourth-class	139	0.52	121	0.45
FQ grade was less than yield grade	2654	9.92	3397	12.69
The same	20913	78.15	19878	74.29
Total	26758	100.00	26758	100.00

^a^ Proportion was calculated as the percent of the FQ grade areas compared to the total farmland.

Abbreviations: FQ, Farmland Quality; GCA, gray correlation analysis; AHP, analytic hierarchy process.

As shown in Figs 6 and 7, the area where FQ grade based on the GCA and AHP methods was more than the yield level was of 3191 ha and 3483 ha, reaching 11.93%, 13.02% of the total farmland, respectively. For these areas, the potential productivity may not be fully tapped. In these regions the standard advice is to increase agricultural investment, optimise the land layout, and improve the field irrigation and drainage systems, etc. [34,35]. The area where FQ grade based on the GCA and AHP methods was less than the yield level was of 2654 ha and 3397 ha, 9.92% and 12.69% of the total farmland, respectively. These areas were mainly in central-southern plains of alluvial fan and plain area with superior natural conditions, more convenient agricultural infrastructure, and sufficient agricultural investment [39,40]. According to Table 1, the two FQ evaluation tests were both good in the study site, and the evaluation results were acceptable.

However, dissimilarities in evaluation purposes, processes and indicators systems for the evaluation of FQ and crop output should not be ignored. For example, the FQ levels based on GCA and AHP are both the measures of potential FQ, while the yield grade reflects the actual capacity of agricultural management, climatic effects and other factors [26,35]. Meanwhile, as FQ evaluation index selection and qualitative index weight determination depends on experts, there are some inevitable deviations in the calculation process. For crop yields, there is lower accuracy in the spatial interpolation of yield maps. Although there are differences between the two evaluation results, the crop yield is still believed to be the most reasonable and reliable test reference [41,42].

Through overlaying the two kinds of map and topologically analysing their consistency rate, this rationality test method proposed for evaluating FQ results would be more quantitative and practical than the usually subjective judgment. To apply this method, thorough investigations to ensure sample accuracy and representativeness are needed. For unacceptable evaluation results, it is necessary to improve indexes, fit model functions or re-calculate index weight, etc. Further research is needed to improve the data analysis methods and the check standard’s rationality, and to revise the evaluation process.

## Conclusions

This study employed GCA to evaluate FQ and analysed the consistency of the results with the traditional AHP. The results of the study area, Xiuwu County, Henan province, China showed the area consistent rate of the two evaluation methods was 84.68%, the evaluation unit consistent rate was 87.39%, and the spatial distribution was equivalent. The evaluation results of the two methods were relatively approximate.

The rationality check procedure of FQ evaluation results in this study was as follows. First, we overlay the crop yield map and FQ evaluation map. Then, we topologically analyse the consistency rate for each grade in the same spatial position. Last, we examine the consistency and determine whether to adopt the evaluation results. FQ evaluation results in the study region was better and acceptable, with the area consistent rate of evaluation results by GCA and crop yield 78.15%, and that by AHP and crop yield 74.29%. Compared with AHP, the GCA results more realistically reflected the land capacity. In brief, compared with the qualitative, more subjective and poor maneuvrability of the existing check measures for FQ evaluation results, the method put forward in this paper would be more precise and objective. However, further studies are required to facilitate its application.

## Supporting Information

S1 Data The relevant data used for all figures. (RAR)
